# Exploring dialectical behaviour therapy clinicians’ experiences of team consultation meetings

**DOI:** 10.1186/s40479-018-0080-1

**Published:** 2018-02-27

**Authors:** Cian Walsh, Patrick Ryan, Daniel Flynn

**Affiliations:** 1 Cork Mental Health Services, Health Service Executive, Psychology Department, Inniscarrig House, Western Road, Cork, Ireland; 2 Department of Psychology, University of Limerick, Limerick, Ireland; 3 Cork Mental Health Services, Health Service Executive, Block 2, St Finbarr’s Hospital, Cork, Ireland

**Keywords:** Dialectical behaviour therapy, Implementation, Team consultation, Borderline personality disorder

## Abstract

**Background:**

This article presents a detailed idiographic analysis of Dialectical Behaviour Therapy (DBT) clinicians’ experiences of team consultation meetings. DBT is an evidence-based psychological intervention with a demonstrated efficacy in the treatment of borderline personality disorder (BPD). Team consultation meetings encompass one of the primary components involved in this treatment model; where DBT clinicians regularly meet to discuss client work and enhance further learning. The present study’s aim was to assess what are DBT clinicians’ experiences of the consultation meeting component and whether it is useful or not.

**Method:**

Semi-structured interviews were completed with 11 DBT clinicians (nine females, two males) from three different consultation teams. The research project utilised an interpretative phenomenological analysis (IPA) framework. Audio-recorded interview data was analysed using this framework.

**Results:**

Four superordinate themes emerged from the interview data, which included ten subordinate themes. The superordinate themes focused on: (1) the acquisition of DBT technical knowledge and other MDT related expertise (2) participants’ emotional experiences of DBT and consultation meetings, and how this can evolve over time (3) the underlying processes that occur in the consultation team including the development of a team bond and the impact of membership changes and (4) the largely consistent and reliable nature of consultation meetings and how they help maintain clinician motivation.

**Conclusions:**

Team consultation meetings were found to be supportive; playing an important role in maintaining clinician motivation through the availability of team support, opportunities to reflect and learn, and assistance in regulating emotions. Challenges arose in relation to team membership changes and acclimatisation to the type of feedback utilised in team consultation. The study’s implications for practise are considered.

## Background

DBT is the primary evidence-based treatment for BPD. It has the highest number of randomised controlled trials (RCTs) investigating its efficacy in treating this disorder [1]. Both the UK Department of Health [2] and the American Psychiatric Association [3] advocate it as one of the leading treatments for BPD, and recent research supports its application in community settings [4].

DBT consists of five treatment components: DBT skills training, individual psychotherapy, phone coaching, case management and DBT team consultation. Ancillary treatments may also play a role alongside these components. For example, pharmocotherapy, day treatment, acute hospitalisation or the attendance of nonprofessional groups such as Alcoholics Anonymous might also be undertaken [5].

Team consultation consists of a weekly meeting between DBT therapists, with the aim of keeping therapists motivated and competent in their treatment of individuals with BPD. Despite the growing evidence-base supporting DBT in the treatment of BPD, there is a noticeable dearth of literature examining the five specific components that comprise the DBT model [6].

Chapman & Linehan [7] note how the consultation team act as a micro-level ‘community’ of providers, where clinicians agree to utilise a dialectical philosophy. This involves a belief that there is no absolute truth, that when opposing views arise, the team look to synthesize these viewpoints rather than looking for a singular truth [5]. In addition, there is less focus on the client’s difficulties and more emphasis put on discussing the therapist’s behaviour within the client-therapist interaction [8]. This discussion happens in a dialectical fashion and is posited to lead to modifications in how the therapist treats their client. Consequently, a transactional system is developed whereby the team influences the therapist’s treatment of their client and the client influences the team’s focus on what therapist behaviour to shape and how the therapist’s motivation should be maintained [8].

Consultation meetings are guided by a set of agreements that suggest how clinicians should interact with each other in consultation and are used in conjunction with a meeting agenda. Swales [9] suggests that three of the agreements are particularly important in maintaining an appropriate learning and supportive atmosphere in consultation: the ‘phenomenological empathy’, ‘fallibility’ and ‘dialectical’ agreements. With the ‘phenomenological empathy’ agreement clinicians search for interpretations of patients’, one’s own and other team members’ behaviour from a non-judgemental and empathic perspective. Using the ‘fallibility’ agreement clinicians acknowledge that all consultation members are susceptible to potential mistakes and agree to let go of defensive stances, when accused of making errors, to help synthesize opposing viewpoints. Finally, with the ‘dialectical agreement’ clinicians acknowledge that there is no absolute truth and clinicians are encouraged to look for the truth in conflicting opinions to help achieve the synthesis of both positions. The overall aim of the agreements is to help reduce and resolve various types of conflict that may occur when people work together in groups [10].

Although there is little empirical research highlighting the existence of conflict in consultation, Simons [11] illustrates how during their initial experiences of consultation a clinician might not always listen to feedback or could become defensive when he did not agree with the evaluation of his practice. In Simon’s example, a non-defensive approach to feedback appeared to develop when the person started to respect different viewpoints from the team. This was due to the shared philosophy of the therapy work; how members could take dialectical positions on a clinician’s approach and thus provide alternative viewpoints.

Swales [12] argues that the various RCT evidence supporting the use of DBT needs to be considered more carefully when DBT is applied in routine clinical settings. She notes that there is a heavy emphasis on training and proper supervision of therapists in these research studies, which might not be available in many standard health care services. In addition, Swales [9] notes that the reallocation of a minimum of one and a half days from clinicians’ regular health service work week to DBT is the first significant organisational challenge when implementing a DBT programme. The one and a half days is for the intervention delivery: time for individual and group DBT work, as well as other related DBT tasks, and 2 hours per week to the DBT consultation component [9].

Swales, Taylor and Hibbs [13] examined relevant implementation factors in DBT programmes in the UK. Their results suggested that the highest programme cessation rates tended to be in the second and fifth years of DBT programme duration. The three primary reasons for the cessation of a programme were lack of organisational support, high staff turnover and insufficient time allotted to the delivery of the programme. Similarly, research by Carmel, Rose, and Fruzzetti [14] highlighted that the time commitment of DBT and a lack of reduction in other work commitments was a significant challenge to the success of DBT programmes. Additional implementation challenges included a lack of administrative support and difficulties pertaining to staff turnover [14]. DBT requires a significant amount of training, which can place added pressure on programme sustainability; particularly in relation to clinicians leaving a programme and the need to recruit new members to the team.

Koerner [15] suggests that resource constraints mean teams may struggle to implement a full treatment protocol or deliver a complete implementation of a consultation team or a phone coaching programme. In this regard, Dubose, Ivanoff, Miga, Dimeff, and Linehan [16] noted the absence of team consultation in a small number of DBT programmes. In a survey of 78 teams, approximately 10% of teams did not have regular team consultation and approximately 6% reported that team consultation meetings never occurred [16]. While it is unclear what effect reduced hours or lack of consultation meetings might have on such DBT programmes, levels of motivation and competency might be adversely affected when we consider the proposed function of consultation meetings. Previous references to DBT team consultation meetings in academic papers tended to be almost entirely descriptive and prescriptive in nature, with a dearth of research assessing the efficacy of team consultation with research evidence or providing any clarification as to whether clinicians’ experiences of it is the same as the proposed theoretical aims of consultation. For example, a literature search using DBT team consultation related terms in the PsychInfo database reveals no journal articles focusing solely on this DBT component. The search did yield the abstract of a Masters dissertation completed by Zahratka [17], which employed quantitative research examining the effect of the consultation team on potential therapist burnout.

In contrast, other DBT articles that mention team consultation tend to do so in a descriptive manner; outlining its proposed function. For example, in Swales’ paper [9] on DBT programme implementation, the author notes that the aim of mandatory weekly consultation meetings is to provide clinicians with supervision and support around their clinical work. In addition, the author proposes that in the training stages the consultation team focus is more on learning, the application of the therapy principles and on the development of case conceptualisation skills. The focus in the consultation team changes towards the supervision of individual cases as the therapists in the team become more competent. However, there is a lack of empirical evidence provided in the paper explicitly illustrating how or if these proposed developments in consultation meetings occur.

Descriptive claims in the other literature also suggest that the purpose of DBT team consultation is to equip clinicians with a means to engage in supervision, provide support and ongoing education, and renewal (e.g. [8, 10, 15, 18]). However, there is a lack of clarity about how clinicians actually experience such aspects of consultation meetings. Similarly, there is a lack of detail on the support experienced from the DBT team in consultation specifically, although Perseius, Kåver, Ekdahl, Åsberg, and Samuelsson [19] suggest a reduction in both stress and the subsequent risk of burnout is partly facilitated by DBT teamwork more generally.

At a more general level, Castonguay & Hill [20] suggest that a greater empirical understanding of therapist effects is needed. Indeed, previous research [21] indicates that differences between therapists more generally can account for 6–9% variance in therapeutic outcomes and Wampold [22] suggests effective therapists exhibit the following characteristics: an ability to form stronger working alliances with different types of clients, strong facilitative interpersonal skills, the expression of more professional self-doubt, and spending more time outside of therapy practicing different therapy skills. Given the variety of disciplines that can make up a DBT consultation team and the proposed focus of DBT consultation meetings on increasing therapist competence and maintaining motivation, it may be useful to explore individuals’ experiences of the consultation meetings more in this regard. The present research aims to provide a detailed exploration of participants’ experiences of the DBT team consultation component; looking at its importance in the model, what benefits clinicians experience from it and some of the challenges that might also take place.

## Method

The current study employed a qualitative, phenomenological, and idiographic approach to explore DBT clinicians’ experiences of team consultation meetings. Consequently, an interpretative phenomenological analysis (IPA) framework was deemed suitable and the procedures recommended by Smith, Flowers and Larkin [23] were followed.

### Participants

Six DBT teams operating in the Republic of Ireland were approached about the research study. Eleven DBT clinicians across three DBT teams agreed to participate. Participants were required to meet two inclusion criteria to be selected for the study: 1) attend regular weekly consultation meetings and 2) have a minimum of 1 year’s experience in their DBT role. The aim of these criteria was to ensure that each participant had sufficient DBT clinical and team consultation experience to reflect upon during an interview. In addition, Swales et al. [13] found that DBT programmes ran an increased risk of failure in the second and fifth years after training. Therefore, the one-year cut-off selection criteria was also chosen to allow for the inclusion of DBT therapists who may potentially be experiencing the types of difficulties which lead to the discontinuation of consultation teams. The participants consisted of nine female and two male participants. The discipline profile consisted of four clinical psychologists, one social worker and six nurses.

### Procedure

The protocol of the present study was approved by a statutorily approved ethics committee that operates as part of the national Health Services Executive. Information about the research study was distributed via email. Team leads were encouraged to inform their respective teams of the research project. In total 12 DBT clinicians expressed interest in the study, but one person fell short of the required one-year DBT experience criteria. An interview schedule was utilised to provide an initial focus to the collection of interview data. This schedule helped explore participants’ involvement in DBT to date, and asked how they experienced the team consultation meetings, what they found beneficial about them and what they experienced as less beneficial. Semi-structured interviews took place face-to-face and lasted between 45 and 60 min. They were audio recorded and transcribed verbatim following the interview.

Each interview transcript was analysed initially on the basis of its own unique terms. As recommended by Smith et al. [23] the analysis involved the development of descriptive, linguistic and conceptual notes for each interview. As IPA is an iterative process that is characterised by an inductive cycle, the analysis took place over an extended period. The researcher read transcripts, left them aside and returned to them over the course of 3 months.

### Validity

To aid the validity of the research several accepted qualitative research criteria were utilised: ‘sensitivity to context’, ‘commitment and rigour’, ‘transparency and coherence’ and ‘impact and importance’ [24, 25]. Sensitivity to context was addressed by interviewing participants in their normal working environment. The interviewer was a trainee clinical psychologist at the time of the research and reflected on this during the analysis and interview stages; seeking clarification and validation of potential interpretations when needed. Further, in relation to commitment and rigour the researcher liaised with another qualitative researcher during the IPA coding process to perform inter-rater reliability checks on a sample of the transcripts. The research team met regularly to discuss the research topic, data collection and analysis thoroughly; working towards a consensus on themes. The researchers regularly re-examined the interview data to ensure that interpretations made from the analysis were reliable and plausible. Although ideally participants would comment on the final analysis, the researchers decided not to proceed with this given the demands on clinicians’ time within a very busy service and the time constraints of the project.

To aid transparency and coherence interpretations of the data are presented alongside illustrative examples (pseudonyms used) from the interviews to help readers gauge their accuracy and assess the impact of the overall study. Impact and importance of the research was considered throughout; selecting a research question that addressed an unexplored area in the academic research and endeavouring to provide some practical utility to clinicians in the write-up, while examining the results in relation to relevant literature.

## Results

The analysis resulted in four superordinate themes and ten subordinate themes; presented in Table 1 below.

**Table 1:** Overview of research themes

Superordinate Themes	Subordinate Themes
Knowledge Acquisition	Model Acclimatisation
	Knowledge Application
	Experience Transfer
Regulation of the Self	Therapeutic Self-concept
	Viewpoint Integration
	Feedback Acclimatisation
Team Processes	Team Bond
	Membership Changes
Motivation and Consistency	Regular and Protected Consultation Time
	Continued Motivation

### Knowledge acquisition

Consultation enabled all clinicians to develop their DBT knowledge and enhance learning in a range of different ways. The following theme highlights the learning experience in relation to model adherence and knowledge acquired from members of the consultation group.

#### Model acclimatisation

Consultation provided a platform to both monitor and learn about the DBT model as well as assist participants in adhering to underlying DBT principles. This was partly achieved by using consultation to share resources, fine-tune DBT materials and discuss elements of the model with other members. However, the experience of learning about the model in consultation appeared initially to be an unsettling one for most participants. There was often a sense that participant anxiety was observed in and shared by fellow members in consultation.

 “I remember the first consult we went to … the language in DBT is quite specific and even the language around consult and things like leader and observer and all this kind of stuff. It felt really strange.” (Deirdre).^1^

There was a distinction in the interview data between experiences of founding members versus experiences of members who joined an established team. Although both expressed anxiety around the acclimatisation to the DBT model, founding members also had the added task of learning how a consultation is operationalised initially.

“I think we definitely have … come a long way with that, ‘cause I mean we started with like literally taking out the book, what are we supposed to be doing now.” (Eleanor).

For many of the participants, acclimatisation to the model was facilitated by a growing familiarity with the underlying principles of the DBT philosophy. Indeed, the consultation team’s ability to facilitate learning and confidence was aided by the regular revision of the DBT agreements, where one agreement would routinely be revisited during each session. While gaps in theoretical knowledge often existed for participants early on, many found the underlying tenets of DBT, in relation to fallibility and the essence of truth, liberating the more they learned about the agreements in consultation. The resulting effect was to reduce the pressure to always be right about the model and how the work should be completed.

“I think the assumptions about you know there being no absolute truth and we’re all fallible and everyone is doing their best but must try harder and all the rest of it… I think it kind of frees us up to make those mistakes” (Frances).

#### Knowledge application

For most participants, the knowledge acquired about the model (including the various DBT skills and principles, as well as the various consultation roles) was reinforced through the active practicing of model components in consultation. Consultation was seen as a useful platform for learning when applying the DBT model to client work; in particular when discussing client issues raised by other team members.

“…if it’s not my client I’m more likely to be better able to think about the DBT principles and to think in behaviourally specific terms.” (Frances).

Consultation provided a space for clinicians to experience the aspects of the DBT model directly. This was particularly relevant to the DBT skills clinicians teach their own clients in other DBT modes i.e. individual client sessions, phone coaching and skills groups.

“Every kind of skill would come up in the consult. Again, I think a lot of emotional regulation stuff…just kind of looking at the emotion, …observing what’s driving the emotion, looking at the function of the emotion.” (Hannah).

#### Experience transfer

In consultation team members often bring with them expertise related to their respective disciplines. Participants found the mix of professions in consultation extremely helpful in gaining knowledge in other relevant areas. In this respect consultation represented a useful opportunity to draw upon the expertise of other disciplines, not just in terms of member knowledge about DBT theory. For example, participants spoke of gaining knowledge around client medication, child protection information and physical injury or specific psychological learning in relation to eating disorders and addiction.

“You can kind of be with a client and you know maybe have one of their target hierarchies could be you know issues with drink or alcohol. And then you can lean towards something he [the addiction counsellor] may have said … and it can kind of shape you.” (Hannah).

Another aspect of experience transfer relates to the acquisition of knowledge from client dilemmas and issues brought to consultation. Although other clinicians might not currently be experiencing the same type of issue, listening to issues creates a type of reservoir of knowledge to draw upon at a later stage.

“… I’ll often have a situation going on even in phone coaching or… with somebody, anyway I’ll think of maybe an experience I’ve heard from somebody else in consult.” (Eleanor).

As one participant suggests “it goes in cycles”*,* where learning acquired from a previous consultation becomes relevant to your own work at a later stage.

### Regulation of the self

This superordinate theme concerns the emotional impact of the client work experienced by participants and the role consultation plays in supporting this phenomenon. The theme examines how emotion is validated, how awareness can be developed in such circumstances and the emotional impact associated with feedback in consultation.

#### Therapeutic self-concept

Participants often referred to the emotional impact of working with clients with BPD. Given the demands in terms of the learning element and the emotional impact of the client work, various emotions such as frustration, doubt and anxiety arose for different individuals in how they completed their work as DBT clinicians. Overall, consultation interaction helped most participants regulate their emotions through the validation of emotion; noting the difficulties associated with particular client issues and how this can cause stress and anxiety in how they perceive themselves and their therapeutic work.

“What was helpful in consult was that I was allowed to express the emotional experience that I was having … and … that again the team validated what was valid in that.” (Ciaran).

Participant experiences of consult also highlighted how a more directive approach was utilised to aid therapeutic self-concept. Aspects such as language and contingencies were questioned or suggested; helping to challenge therapists' viewpoints.

“‘Cause we can go down the road ‘Oh I’m absolutely useless’ … and I think the group helps to ground you and helps you to think about the language that you are using.” (Margaret).

Another aspect of therapeutic self-concept was around the choice of action selected by the clinician. Many participants found the team would often validate their choice of action, which appeared to have a regulating effect in reducing anxiety or doubt over their personal approach to the work.

“It’s very helpful as well when they say ‘Yeah that’s exactly what I’d have done as well’.” (Jennifer).

Participants mentioned structural elements of consultation and how they encouraged clinicians to reflect on their current therapeutic self-concept. This was primarily in relation to the routine questions posed at the start of consultation, which focus specifically on the clinician rather than the client. Particularly pertinent was the questioning of whether any therapists were in a state of ‘high emotion’. For most participants, this had the effect of providing space to share high emotion, as well as validating the fact that the work can be very demanding.

#### Viewpoint integration

This theme concerns the evolution of consultation members’ viewpoints or insights and how they choose what actions to take in their DBT work. What differentiates the theme from knowledge acquisition is the interpretative quality to these insights as well as how the team input helps provide new perspectives.

Participants acknowledged how they may have the academic and technical knowledge but may still experience ‘blind spots’ during the work. This was not necessarily due to high emotion in the clinician, but rather due to a simple oversight or the large number of variables involved when working with complex clients. However, developing new insights was not simply a case of receiving suggestions from the team. Interview data highlighted that when new perspectives were brought into the participant’s awareness, they must then still decide on what action to take, sometimes employing DBT principles to do so.

“And you can take all elements and put it together rather than you know it’s not that one person is saying ‘The way that I do it is right’.” (Hannah).

#### Feedback acclimatisation

Most participants did not acclimatise to the feedback process in consultation immediately. The process of feedback acclimatisation was aided by a growing familiarisation with the DBT philosophy and model underpinning the way feedback is delivered, such as the use of descriptive language rather than judgemental language when providing feedback, not treating fellow members as fragile and acknowledging the diversity of member views. However, some participants’ early experiences of feedback in consultation were particularly negative, affecting clinician confidence as well as motivation to attend consultation.

“… I was allergic to going to consult for maybe two weeks after that ...” (Jennifer).

“…you can feel like you’re getting bombarded sometimes. And also I think it feels like [to] me it can feel …you’re … being undermined” (Peter).

Above, Jennifer is referring to feedback received in consultation after providing details on a difficult client session. What made the feedback hard for her was partly due to how she was relatively new to the consultation team; that many of the people in the meeting had roughly two years more experience of DBT. For Jennifer it felt like her consultation colleagues were saying “Oh I wouldn’t have done that” or “Oh I don’t know if that was the best thing to do”. The feedback was perceived as invalidating to her, at a time where she felt she did the best she could and was looking for next steps to fix an issue rather than feedback on what she had done already.

In contrast, Peter had more experience in his particular consultation team, but as a psychologist he was used to a different type of feedback in clinical supervision. For him the feedback in his consultation meeting felt very direct and almost competitive at times. He felt when feedback in the meeting was being given it was less focused on the “relationship type stuff” that he would have in regular psychology supervision.

Often acclimatisation to how feedback is delivered in consultation led to a growing appreciation for it. Essentially, what appeared to develop for participants was an ability to focus more on the function of the feedback rather than the perceived manner it was delivered.

“But I think once I got used to it [the feedback] certainly it became a lot easier and I grew to value it.” (Frances).

### Team processes

This theme is evidence of participants’ experiences of some of the underlying processes that occur in the consultation team. These concern the development of a team bond over time as well as the impact membership changes have on participants’ experience of consultation.

#### Team bond

Most interviewees were positive about the team experience; referring to a sense of comfort and support. Often it appeared that a team bond in consultation was important in developing a sense of safety and in aiding the smooth running of the meeting. Participants reported that they were more likely to learn more from the feedback process and support if they felt the relationship was strong within the team.

Often evidence of team bonding manifested itself in a sense of joviality and fun at different stages during meetings. Experiences of fun and joviality described in consultation had an important function; namely to help participants and their team manage the stresses of the work. Also, there was a shared understanding amongst participants around how only fellow consultation members fully understand the nature and demands of the work.

“Sometimes more than other times … humour is a really good I suppose way of diffusing the intensity of those experiences.” (Ciaran).

“None of the community mental health team I don’t think understand what you’re doing and that’s why the consult is really important. Because we all understand the role, the function. And we’re all working very hard.” (Niamh).

Experiences within DBT consultation were reported as being influential in developing outside support with team members. Indeed, in terms of the depth of team bond, perhaps the most potent illustration was the presence or absence of “consult outside of consult”.

“I’d ring people if like even at night-time …. like you wouldn’t really dream of doing that with other colleagues, unless I was friends with them do you know… and I think that’s all learned from consult…” (Jennifer).

#### Membership changes

All participants referred to changes in membership of the consultation team. Participants’ references to team membership changes were mainly in relation to new members joining. It also pertained to the impact of team members leaving due to promotions, changes of location and career breaks.

The reaction to new consultation team members was mixed. Some participants recognised the benefit and necessity of new clinicians joining the group in terms of new knowledge and different ways of working. However, for some other participants the difficulty with new members was their apparent impact on participants’ ability to be open in consultation, as well as the potential effect on existing team cohesiveness.

“…I noticed again when new members would join the team that people would become a little bit less comfortable again understandably. And express a little less until again built up that comfort [with] people.” (Ciaran).

Participants who joined established teams often did so along with other new members; with whom they had completed their training. Therefore, they too had their own subgroups and own level of comfort with each other prior to joining a bigger consultation team. New members could potentially identify with each other’s anxiety around how established members had more experience than them.

“…it was anxiety provoking at the start… thinking these people are more advanced than me … initially being a bit I suppose apprehensive about talking.” (Hannah).

### Motivation and consistency

This theme represents the largely consistent and reliable nature of consultation and how it acts as a motivator for clinicians.

#### Regular and protected consultation time

Consultation acted as an anchor or milestone during the work-week, representing a place of both mental and physical refuge from the stresses and strains of work for most participants. Having a regular consultation time provided many clinicians with a greater sense of security in their clinical work.

“In terms of knowing you’re not kind of stuck with this on your own, that within the week you’re going to be back talking with your peers about it.” (Deirdre).

From a systems perspective, consultation was seen as a useful timetabled structure in the week to help buffer demands from other work sources.

“It’s great to be able to say …I won’t be in consult until Tuesday so…there’s not pressure on you to give your own sort of personal opinion or any answers to anything.” (Eleanor).

However, some participants found that time constraints during consultation were a growing issue. Firstly, four participants reported that business meetings were a significant factor in relation to consultation time infringement; how they left “very little time for … the actual full consult” (Geraldine); thus eroding the consistent nature of consultation. Secondly, the number of consultation members was a growing concern for some as the prospect of more new members joining the team created some unease around having sufficient time to discuss their clients.

#### Continued motivation

There was an overwhelming consensus that consultation was a necessity in terms of motivating clinicians to work over the long-term. When asked how they would fare in the absence of consultation, each participant stated they would not feel safe in continuing; with the majority stating that they would in fact *not* continue to practice in its absence.

“I don’t think I could stay in DBT if consult wasn’t available… I don’t think I’d be able to survive doing it…” (Lisa).

In some cases, consultation was even perceived as a motivational reward for the hard work invested in other aspects of DBT.

“… I think the consult is a big piece of what sort of is the pay-off of that [the hard work] … if I didn’t have consult I’d feel like I’m putting a lot of my time and energy into this and I could actually go away and do my day-job…” (Deirdre).

Participants referred to how supervision helped to answer questions that were not resolved in consultation. In addition, some participants referred directly to how supervision also plays a role in supporting the continued motivation of clinicians. This was particularly relevant in relation to maintaining consistent adherence to the consultation model, helping teams to return to optimum performance levels.

“[Our supervisor] said to us … ‘Ye need to get back in gear…ye need to start … doing your consult’ you know have… your observer your chairperson you know the mindfulness. Once we went back in and started doing that it was actually very powerful.” (Lisa).

## Discussion

The present study sought to assess a sample of DBT clinicians’ experiences of the consultation meeting component and whether this component is useful or not. Of related importance was to seek evidence if the theoretical functions of the consultation team might be supported or not. The limited literature on DBT team consultation suggested some potentially relevant aspects pertinent to clinicians’ experiences of these meetings. These included the proposed learning or training environment created by the consultation team and the team’s ability to maintain model adherence and the continued effectiveness of clinician work (e.g. [5, 8, 26]). Clinicians’ narratives in the present study supported these claims, whereby clinicians learned about the DBT model in consultation through the sharing of resources, discussing DBT learning points and fine-tuning materials together. Further, the present research provided an explicit deconstruction of the types of learning challenges involved in consultation; including the acclimatisation to DBT terminology and principles, as well as the generalisation of DBT skills from consultation.

Regarding how clinicians learn in consultation, Kolb’s [27] learning cycle model presents as a relevant framework as its various components are suggested in the described experiences of participants in the present study. Cited as the most influential learning style model [28], Kolb’s model focuses on the experiential learning rather than on fixed learning traits [29]. It is based on four learning modes: concrete experience, reflective observation, abstract conceptualisation and active experimentation. In the concrete experience stage, the individual or team encounters a new experience or reinterpretation of an existing experience. The reflective observation stage involves reflecting on that experience, while the abstract conceptualisation stage is characterised by the formation of new ideas or potential modifications of existing abstract ideas. Finally, the active experimentation stage is where the individual applies any insights and ideas in the planning for the next experience.

There were plenty of references to how participants brought concrete experiences or dilemmas to consultation, which were subsequently discussed by the team. While Kolb’s [27] cycle is a useful way of conceptualising how aspects of learning are assimilated in consultation, the philosophical principles of DBT arguably make this process less clear-cut. Participants referred to an underlying philosophy in DBT, whereby there is no one ‘truth’ and that various perspectives will have their own level of validity for any given individual. This in turn may diminish the abstract conceptualisation stage of Kolb’s cycle, where existing abstract concepts possessed by the clinician are not necessarily modified but how other perspectives are flexibly incorporated instead.

A common narrative amongst participants was the emotional impact of working with individuals with BPD. This echoed the findings of previous research (e.g. [30, 31]), which highlighted the emotional dysregulation often associated with working with this client group. While participants in the present study often experienced emotion in their DBT work, the results also highlighted participant ability to regulate themselves through the use of consultation, which was fundamentally identified as a validating and supportive space despite occasional difficulties with feedback from team members.

Difficulties related to feedback acclimatisation may explain why team members potentially refrain from disclosing regularly until they are orientated and experienced in the feedback process and assimilate learning around the DBT team consultation principles. Although Swales [9] claims consultation agreements facilitate a non-defensive attitude, it appeared that some clinicians felt vulnerable during their earlier experiences of consultation meetings. Research has previously noted such feedback difficulties [11], and the current study has generated evidence to suggest that discomfort with feedback may be due to feeling judged by others initially or due to the volume of suggestions from team members, which was overwhelming at times. Despite such challenges, the clinicians involved in the present study highlighted their overwhelming desire for consultation meetings in order to maintain their motivation to continue engaging in DBT. BPD can often present co-morbidly with other mental health disorders (e.g. [32, 33]). In the present study, the variety of professional experience in the consultation team helped clarify potentially more complex presentations; such as gaining relevant addiction knowledge from addiction counsellors or gaining knowledge from psychologists in the team who had expertise on eating disorders. This poses interesting questions about whether a broader range of experience needs to be considered in consult team make-up.

Throughout participant narratives the importance of some of the DBT therapist consultation agreements formulated by Linehan [5] were highlighted; in particular, the ‘phenomenological empathy agreement’, the ‘fallibility agreement’ and the ‘dialectical philosophy agreement’. The fallibility agreement appeared important in relation to potentially influencing clinician disclosure more readily in consultation. Although there is a lack of literature examining clinicians’ experiences of DBT consultation meetings, research in other fields indicates that clinicians may find it harder to disclose in a group as opposed to individual supervision settings (e.g. [34]). Participants in the present study did not express great concern with such disclosure, although some participants reported initial reticence when new members joined the consultation team.

The ‘team bond’ theme supported academic literature descriptions of the consultation team as a ‘community’ (e.g. [7]). While the main function of consultation meetings is to ensure clinician effectiveness [5], the findings of the present study suggest that the quality of the team dynamic is very important in delivering this objective. Here the social aspect present in some of the consultation teams was important, as well as the presence of ad-hoc peer support outside of sessions. Carmel et al. [14] and Swales [9] note the organisational challenges faced by DBT clinicians with respect to giving a day and a half to DBT each week; including a half-day for consultation. Generally, clinicians in the present study appreciated having a consistent time and place to meet for consultation and prioritised it over other meetings and commitments, thereby creating the dynamic for team bonding to be both initiated and maintained.

Carmel et al. [14] and Swales et al. [13] report the challenges associated with high staff turnover; suggesting the importance of new team members joining in order to extend the survival of a DBT programme. In the present study, the impact of staff turnover in relation to the general consultation experience was reflected in the loss of certain multidisciplinary expertise or a supportive characteristic of a former team member. Expanding upon the DBT implementation literature, however, the participant accounts in the present study highlight more specifically how new members not only sustain DBT programmes from a numbers perspective but how new members can help rejuvenate adherence levels and advance learning by sharing up-to-date information from their more recent DBT training. From a different perspective, the present study also highlighted potential challenges to new members joining consultation. This included how some clinicians found it difficult to adjust to new members joining team consultation as they had grown comfortable with the level of trust and team bond that had developed previously.

In their research examining implementation of DBT programmes, Swales et al. [13] suggest that programmes with more than six team members and who have less than one day per week allocated to DBT, might benefit from reducing staff to provide individual clinicians with sufficient time to discuss client issues in weekly consultation meetings. The impact of the number of members was reflected in the present study, wherein some participants feared that if membership numbers increased they might not have sufficient time to express and discuss their individual client issues. Similarly, participants referred to the impact of occasional DBT business meetings and how these can infringe upon regular consultation time and the discussion of client and clinician issues.

### Limitations and strengths

The main limitation of the study concerns its restricted generalisability. Clinicians who expressed an interest in the study may well have been more appreciative of consult compared to other DBT clinicians who did not agree to participate in the study. One of the three consultation teams accounted for seven of the eleven participants, which may potentially highlight this suggestion. Given the idiographic focus of IPA, however, the aim of the research was to explore individual experiences, and therefore the study did not seek to make general claims about wider populations and other consultations.

A major strength of the study is how it addressed a noticeable dearth of research on team consultation. Further, the employment of a qualitative approach, and the use of IPA to inform the research focus and analysis, was particularly useful in developing a narrative richness around clinicians’ experiences of team consultation meetings. Although idiographic in its focus, the triangulation of the various participant accounts and related reflection on the analysis experience led to robust superordinate and subordinate themes.

Future research might employ a similar qualitative approach to explore clinicians’ experience of DBT supervision or perhaps clinicians’ experiences of team consultation meetings in DBT programmes developed for different populations. Further, quantitative research might examine the role of team makeup (e.g. skill or discipline mix, years experience as a mental health practitioner) in relation to model effectiveness.

## Conclusions

The current study illustrates the main experiential features of DBT team consultation meetings; highlighting evidence that supports the theoretical functions that the consultation team component has within the DBT model. However, the results also highlight the presence of an acclimatisation process when first joining a DBT consultation team, whereby clinicians are often faced with challenges of new theoretical learning, receiving feedback on how to adherently apply theory to practice while developing a sense of cohesion with the consultation team. This process differs in terms of whether a therapist is joining an existing consultation team or forming a new consultation team. Although the delivery of team member feedback in consultation is guided by DBT team consultation agreements, feedback can appear overly direct and be potentially misconstrued by newer members as critical rather than constructive guidance on becoming more model adherent. Members’ perception of feedback in this regard might possibly affect motivation levels.

Founding members of a new consultation team might also benefit from more support to help orientate to the role and structure of consultation. More generally, specific information around the benefits and challenges associated with consultation might be shared with prospective DBT trainees to help better prepare them for this model component. This might be considered within the context of training delivery, with a great focus on experiential learning (e.g. role-play and practice in giving and receiving feedback in model adherence). Furthermore, individuals and teams may benefit from supervision to move from understanding consultation agreements theoretically to embodying the agreements in their consultation practice. From the perspective of the clinicians in the study ad-hoc peer support appeared to be a useful resource. The availability of peer support outside of allotted DBT consultation hours appeared indicative of stronger team relationships and was particularly useful in regulating clinician emotion. This type of support might benefit from becoming a structured component of the DBT setup as it does appear to be a helpful coping mechanism when working in such a challenging area. However, the presence of peer support, or consultation teams more generally, does not necessarily mean that this type of support actually improves team relationships or that consultation teams actually help the clinician to work in DBT. Future research might test this by means of a dismantling study i.e. examining the efficacy of DBT with or without the consultation component. Where some studies have indicated the absence of team consultation [16], the current study highlights the value of consultation meetings in terms of gaining knowledge, helping regulate one’s responses to working with high risk clients and continued motivation and support for adherent and sustained DBT clinical practice.

## Abbreviations

BPD: Borderline Personality Disorder
DBT: Dialectical behavior therapy
IPA: Interpretative Phenomenological Analysis
RCT: Randomized controlled trial

## Footnotes

1 Pseudonyms were used for all participant quotes

## References

[1] Stoffers JM, Völlm BA, Rücker G, Timmer A, Huband N, Lieb K (2012). Psychological therapies for people with borderline personality disorder. Cochrane Database System Rev.

[2] Snowden P, Kane E (2003). Personality disorder: no longer a diagnosis of exclusion. Psychiatr Bull.

[3] American Psychiatric Association (2000). Practice guideline for the treatment of patients with borderline personality disorder.

[4] Flynn D, Kells M, Joyce M, Corcoran P, Gillespie C, Suarez C, Cotter P (2017). Standard 12 month dialectical behaviour therapy for adults with borderline personality disorder in a public community mental health setting. Borderline Personality Disor Emo Dysregul.

[5] Linehan M (1993). Cognitive-behavioral treatment of borderline personality disorder.

[6] Rizvi SL, Steffel LM, Carson-Wong A (2013). An overview of dialectical behavior therapy for professional psychologists. Prof Psychol Res Prac.

[7] Chapman AL, Linehan M, Zanarini MC (2005). Dialectical behavior therapy for borderline personality disorder. Borderline personality disorder.

[8] Lynch TR, Chapman AL, Rosenthal MZ, Kuo JR, Linehan MM (2006). Mechanisms of change in dialectical behavior therapy: theoretical and empirical observations. J Clin Psychol.

[9] Swales MA (2010). Implementing DBT: selecting, training and supervising a team. Cog BehavTher.

[10] Swales MA, Heard HL (2009). Dialectical behaviour therapy: distinctive features.

[11] Simons MK (2010). Client and clinician experiences of dialectical behaviour therapy: a discourse analysis. (doctor of clinical psychology PhD thesis), Massey University.

[12] Swales MA (2010). Implementing dialectical behaviour therapy: organizational pre-treatment. Cog Behav Ther.

[13] Swales MA, Taylor B, Hibbs RA (2012). Implementing dialectical behaviour therapy: Programme survival in routine healthcare settings. J Ment Health.

[14] Carmel A, Rose ML, Fruzzetti AE (2014). Barriers and solutions to implementing dialectical behavior therapy in a public behavioral health system. Adm Policy Ment Health Ment Health Serv Res.

[15] Koerner K (2013). What must you know and do to get good outcomes with DBT?. Behav Ther.

[16] Dubose AP, Ivanoff A, Miga E, Dimeff L, Linehan MM. DBT teams in training 2008–2011: implementation follow-up in 2012. Paper presented at the 2nd biennial conference: solving implementation research dilemmas. Seattle: WA; 2013. https://societyforimplementationresearchcollaboration.org/conference-2-may-2013/.

[17] Zahratka C (2010). Dialectical behavior therapy: the effect of the consultation team on potential therapist burnout. Diss Abstr Int.

[18] Mayer-Bruns F (2013). Skills für therapeuten in DBT-teamarbeit und-supervision. Psychothérapies.

[19] Perseius KI, Kåver A, Ekdahl S, Åsberg M, Samuelsson M (2017). Stress and burnout in psychiatric professionals when starting to use dialectical behavioural therapy in the work with young self-harming women showing borderline personality symptoms. J Psychiatr Ment Health Nurs.

[20] Castonguay LG, Hill CE (2017). How and why are some therapists better than others?: understanding therapist effects.

[21] Wampold BE (2001). The great psychotherapy debate models, methods, and findings.

[22] Wampold BE (2015). How important are the common factors in psychotherapy? An update. World Psychiatry.

[23] Smith JA, Flowers P, Larkin M (2009). Interpretative phenomenological analysis: theory, method and research.

[24] Yardley L (2000). Dilemmas in qualitative health research. Psychol Health.

[25] Yardley L, Smith JA (2008). Demonstrating validity in qualitative psychology. Qualitative psychology: a practical guide to research methods.

[26] Swenson CR, Torrey WC, Koerner K (2014). Implementing dialectical behavior therapy. Psychiatr Serv.

[27] Kolb DA (1984). Experimental learning: experience as the source of learning and development.

[28] Kayes DC (2005). Internal validity and reliability of Kolb's learning style inventory version 3 (1999). J Bus Psychol.

[29] Turesky EF, Gallagher D (2011). Know thyself: coaching for leadership using Kolb's experiential learning theory. Coaching Psychol.

[30] Bodner E, Cohen-Fridel S, Iancu I (2011). Staff attitudes toward patients with borderline personality disorder. Compr Psychiatry.

[31] McGrath B, Dowling M (2012). Exploring registered psychiatric nurses' responses towards service users with a diagnosis of borderline personality disorder. Nursing res prac.

[32] Grant BF, Chou SP, Goldstein RB, Huang B, Stinson FS, Saha TD, Pickering RP (2008). Prevalence correlates, disability, and comorbidity of DSM-IV borderline personality disorder: results from the wave 2 national epidemiologic survey on alcohol and related conditions. J clin psychiatry.

[33] Lenzenweger MF, Lane MC, Loranger AW, Kessler RC (2007). DSM-IV personality disorders in the national comorbidity survey replication. Biol Psychiatry.

[34] Webb A, Wheeler S (1998). How honest do counsellors dare to be in the supervisory relationship?: an exploratory study. Br J Guid Couns.

